# The genome sequence of the Mountain Bumble Bee, *Bombus monticola* Smith, 1849 (Hymenoptera: Apidae)

**DOI:** 10.12688/wellcomeopenres.24577.1

**Published:** 2025-07-25

**Authors:** Louise Hislop, Wellcome Sanger Institute Tree of Life Management, Samples and Laboratory team, Wellcome Sanger Institute Scientific Operations: Sequencing Operations, Wellcome Sanger Institute Tree of Life Core Informatics team, Tree of Life Core Informatics collective, Darwin Tree of Life Consortium

**Affiliations:** 1 Independent researcher, Wylam, Northumberland, England, UK

**Keywords:** Bombus monticola, Mountain Bumble Bee, genome sequence, chromosomal, Hymenoptera

## Abstract

We present a genome assembly from a female specimen of *Bombus monticola* (Mountain Bumble Bee; Arthropoda; Insecta; Hymenoptera; Apidae). The genome sequence has a total length of 306.99 megabases. Most of the assembly (90.92%) is scaffolded into 18 chromosomal pseudomolecules. The mitochondrial genome has also been assembled, with a length of 24.12 kilobases.

## Species taxonomy

Eukaryota; Opisthokonta; Metazoa; Eumetazoa; Bilateria; Protostomia; Ecdysozoa; Panarthropoda; Arthropoda; Mandibulata; Pancrustacea; Hexapoda; Insecta; Dicondylia; Pterygota; Neoptera; Endopterygota; Hymenoptera; Apocrita; Aculeata; Apoidea; Anthophila; Apidae; Apinae; Bombini; *Bombus*; *Pyrobombus*; *Bombus monticola* Smith, 1849 (NCBI:txid103936)

## Background

**Figure 1.**
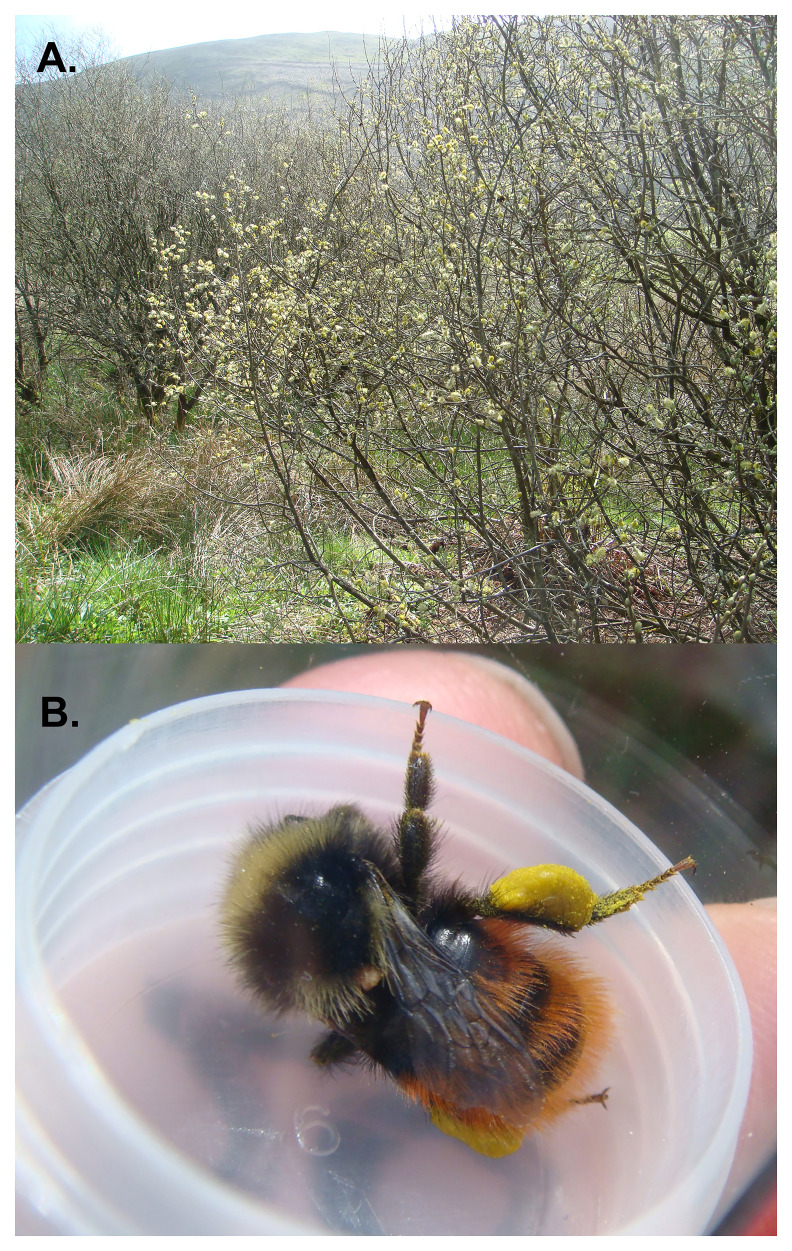
Photograph of the *Bombus monticola* and its habitat.

*Bombus monticola* ( Figure 1), the Mountain or Bilberry Bumblebee is a boreo-alpine species found only in western Europe. In the UK *B. monticola* is found in upland areas in the north and west, but also along coasts in a few areas. Formerly more widespread, it is recorded from Dorset and Cornwall, through Wales and the north Midlands, Pennines in northern England, to the Highlands of Scotland. It is absent from the Scottish islands. First recorded in Ireland in 1974 and possibly expanding its rage there. Strongholds are in Speyside, Dartmoor, Exmoor, Peak District, Welsh uplands, northern Pennines.

The overwintered queens emerge late, often not appearing until April in the south, in May further north or at altitude. Workers follow from May, with new queens and males from late July. Colonies can continue into September or October. It is polylectic, collecting pollen from families Asteraceae, Lemnaceae and Fabaceae ( Else & Edwards, 2018). This species shows a preference for higher altitude heath and moor with a varied landscape including willows, bilberry, heathers, Bird’s-foot trefoil, bramble, Raspberry, Devil’s-bit Scabious, clovers. Nesting occurs underground, predominantly in old rodent burrows. Colonies are small, often with fewer than 50 workers ( Falk & Lewington, 2015). The lifecycle is short, about 3–4 months ( Bees Wasps & Ants Recording Society (BWARS)).

Small for a bumblebee (queens 16–30 mm, workers 11–15 mm) ( Else & Edwards, 2018), this is a short-faced species with a short tongue. The broad queens have two bands of strong primrose-yellow hair on the thorax: a wide band at the front and a narrower fringe on the scutellum at the rear. It has a distinctive orange-red tail that extends considerably further up the abdomen than in other species, beginning at tergite 2. Hairs on the tip of the tail are often pale yellow or whitish. Workers are small versions of the queen. Males are round and fluffy, with the same red tail but more extensive yellow hair on the front of the thorax, top of the head and face. The male genitalia are very similar to those of *B. pratorum*.

It is possibly the host of the parasitic ‘cuckoo’ bumblebee *Bombus sylvestris* Lepeletier ( Edwards & Broad, 2005).

In the IUCN Red List of Threatened Species assessment ( Rasmont *et al.*, 2015), *Bombus monticola* is listed as Least Concern. In Britain this species was not regarded as being scarce or threatened but has now been included on English Nature’s Species Recovery Programme because of the modern evidence of serious decline ( Bees Wasps & Ants Recording Society (BWARS)).

As part of the Darwin Tree of Life Project – which aims to generate high-quality reference genomes for all named eukaryotic species in Britain and Ireland to support research, conservation, and the sustainable use of biodiversity – we present a chromosomally complete genome sequence for *Bombus monticola*, the Mountain Bumble Bee.

## Methods

### Sample acquisition

The specimen used for genome sequencing was an adult female *Bombus monticola* (specimen ID SAN00002545, ToLID iyBomMont4; Figure 1), collected from Carrifran Wildwood, Scotland, UK (latitude 55.3911, longitude –3.3279) on 2022-05-22. The specimen was collected and identified by Louise Hislop (Edinburgh Entomological Club).

### Nucleic acid extraction

Protocols for high molecular weight (HMW) DNA extraction developed at the Wellcome Sanger Institute (WSI) Tree of Life Core Laboratory are available on protocols.io ( Howard *et al.*, 2025). The iyBomMont4 sample was weighed and triaged to determine the appropriate extraction protocol. Tissue from the thorax was homogenised by powermashing using a PowerMasher II tissue disruptor. HMW DNA was extracted in the WSI Scientific Operations core using the Automated MagAttract v2 protocol. DNA was sheared into an average fragment size of 12–20 kb following the Megaruptor®3 for LI PacBio protocol. Sheared DNA was purified by manual SPRI (solid-phase reversible immobilisation). The concentration of the sheared and purified DNA was assessed using a Nanodrop spectrophotometer and Qubit Fluorometer using the Qubit dsDNA High Sensitivity Assay kit. Fragment size distribution was evaluated by running the sample on the FemtoPulse system. For this sample, the final post-shearing DNA had a Qubit concentration of 19.46 ng/μL and a yield of 875.70 ng, with a fragment size of 12.7 kb. The 260/280 spectrophotometric ratio was 1.92, and the 260/230 ratio was 1.72.

RNA was extracted from thorax tissue of iyBomMont4 in the Tree of Life Laboratory at the WSI using the RNA Extraction: Automated MagMax™ *mir*Vana protocol. The RNA concentration was assessed using a Nanodrop spectrophotometer and a Qubit Fluorometer using the Qubit RNA Broad-Range Assay kit. Analysis of the integrity of the RNA was done using the Agilent RNA 6000 Pico Kit and Eukaryotic Total RNA assay.

### PacBio HiFi library preparation and sequencing

Library preparation and sequencing were performed at the WSI Scientific Operations core. Libraries were prepared using the SMRTbell Prep Kit 3.0 (Pacific Biosciences, California, USA), following the manufacturer’s instructions. The kit includes reagents for end repair/A-tailing, adapter ligation, post-ligation SMRTbell bead clean-up, and nuclease treatment. Size selection and clean-up were performed using diluted AMPure PB beads (Pacific Biosciences). DNA concentration was quantified using a Qubit Fluorometer v4.0 (ThermoFisher Scientific) and the Qubit 1X dsDNA HS assay kit. Final library fragment size was assessed with the Agilent Femto Pulse Automated Pulsed Field CE Instrument (Agilent Technologies) using the gDNA 55 kb BAC analysis kit.

The sample was sequenced using the Sequel IIe system (Pacific Biosciences, California, USA). The concentration of the library loaded onto the Sequel IIe was in the range 40–135 pM. The SMRT link software, a PacBio web-based end-to-end workflow manager, was used to set-up and monitor the run, and to perform primary and secondary analysis of the data upon completion.

### Hi-C

***Sample preparation and crosslinking***

The Hi-C sample was prepared from 20–50 mg of frozen head tissue of the iyBomMont4 sample using the Arima-HiC v2 kit (Arima Genomics). Following the manufacturer’s instructions, tissue was fixed and DNA crosslinked using TC buffer to a final formaldehyde concentration of 2%. The tissue was homogenised using the Diagnocine Power Masher-II. Crosslinked DNA was digested with a restriction enzyme master mix, biotinylated, and ligated. Clean-up was performed with SPRISelect beads before library preparation. DNA concentration was measured with the Qubit Fluorometer (Thermo Fisher Scientific) and Qubit HS Assay Kit. The biotinylation percentage was estimated using the Arima-HiC v2 QC beads.

***Hi-C library preparation and sequencing***

Biotinylated DNA constructs were fragmented using a Covaris E220 sonicator and size selected to 400–600 bp using SPRISelect beads. DNA was enriched with Arima-HiC v2 kit Enrichment beads. End repair, A-tailing, and adapter ligation were carried out with the NEBNext Ultra II DNA Library Prep Kit (New England Biolabs), following a modified protocol where library preparation occurs while DNA remains bound to the Enrichment beads. Library amplification was performed using KAPA HiFi HotStart mix and a custom Unique Dual Index (UDI) barcode set (Integrated DNA Technologies). Depending on sample concentration and biotinylation percentage determined at the crosslinking stage, libraries were amplified with 10–16 PCR cycles. Post-PCR clean-up was performed with SPRISelect beads. Libraries were quantified using the AccuClear Ultra High Sensitivity dsDNA Standards Assay Kit (Biotium) and a FLUOstar Omega plate reader (BMG Labtech).

Prior to sequencing, libraries were normalised to 10 ng/μL. Normalised libraries were quantified again and equimolar and/or weighted 2.8 nM pools. Pool concentrations were checked using the Agilent 4200 TapeStation (Agilent) with High Sensitivity D500 reagents before sequencing. Sequencing was performed using paired-end 150 bp reads on the Illumina NovaSeq 6000.

### RNA library preparation and sequencing

Libraries were prepared using the NEBNext ^®^ Ultra™ II Directional RNA Library Prep Kit for Illumina (New England Biolabs), following the manufacturer’s instructions. Poly(A) mRNA in the total RNA solution was isolated using oligo(dT) beads, converted to cDNA, and uniquely indexed; 14 PCR cycles were performed. Libraries were size-selected to produce fragments between 100–300 bp. Libraries were quantified, normalised, pooled to a final concentration of 2.8 nM, and diluted to 150 pM for loading. Sequencing was carried out on the Illumina NovaSeq 6000 to generate 150-bp paired-end reads.

### Genome assembly

Prior to assembly of the PacBio HiFi reads, a database of *k*-mer counts ( *k* = 31) was generated from the filtered reads using FastK. GenomeScope2 ( Ranallo-Benavidez *et al.*, 2020) was used to analyse the *k*-mer frequency distributions, providing estimates of genome size, heterozygosity, and repeat content.

The HiFi reads were assembled using Hifiasm ( Cheng *et al.*, 2021) with the --primary option. Haplotypic duplications were identified and removed using purge_dups ( Guan *et al.*, 2020). The Hi-C reads ( Rao *et al.*, 2014) were mapped to the primary contigs using bwa-mem2 ( Vasimuddin *et al.*, 2019), and the contigs were scaffolded in YaHS ( Zhou *et al.*, 2023) with the --break option for handling potential misassemblies. The scaffolded assemblies were evaluated using Gfastats ( Formenti *et al.*, 2022), BUSCO ( Manni *et al.*, 2021) and MERQURY.FK ( Rhie *et al.*, 2020).

The mitochondrial genome was assembled using MitoHiFi ( Uliano-Silva *et al.*, 2023), which runs MitoFinder ( Allio *et al.*, 2020) and uses these annotations to select the final mitochondrial contig and to ensure the general quality of the sequence.

### Assembly curation

The assembly was decontaminated using the Assembly Screen for Cobionts and Contaminants ( ASCC) pipeline. TreeVal was used to generate the flat files and maps for use in curation. Manual curation was conducted primarily in PretextView and HiGlass ( Kerpedjiev *et al.*, 2018). Scaffolds were visually inspected and corrected as described by Howe *et al*. (2021). Manual corrections included 2 breaks, 24 joins, and removal of 1 haplotypic duplication. The curation process is documented at https://gitlab.com/wtsi-grit/rapid-curation. PretextSnapshot was used to generate a Hi-C contact map of the final assembly.

### Assembly quality assessment

The Merqury.FK tool ( Rhie *et al.*, 2020) was run in a Singularity container ( Kurtzer *et al.*, 2017) to evaluate *k*-mer completeness and assembly quality for the primary and alternate haplotypes using the *k*-mer databases ( *k* = 31) computed prior to genome assembly. The analysis outputs included assembly QV scores and completeness statistics.

The genome was analysed using the BlobToolKit pipeline, a Nextflow implementation of the earlier Snakemake version ( Challis *et al.*, 2020). The pipeline aligns PacBio reads using minimap2 ( Li, 2018) and SAMtools ( Danecek *et al.*, 2021) to generate coverage tracks. It runs BUSCO ( Manni *et al.*, 2021) using lineages identified by querying the GoaT database ( Challis *et al.*, 2023). For the three domain-level lineages, BUSCO genes are aligned to the UniProt Reference Proteomes database ( Bateman *et al.*, 2023) using DIAMOND blastp ( Buchfink *et al.*, 2021).The genome is divided into chunks based on the density of BUSCO genes from the closest taxonomic lineage, and each chunk is aligned to the UniProt Reference Proteomes database with DIAMOND blastx. Sequences without hits are chunked using seqtk and aligned to the NT database with blastn ( Altschul *et al.*, 1990). The BlobToolKit suite consolidates all outputs into a blobdir for visualisation. The BlobToolKit pipeline was developed using nf-core tooling ( Ewels *et al.*, 2020) and MultiQC ( Ewels *et al.*, 2016), with package management via Conda and Bioconda ( Grüning *et al.*, 2018), and containerisation through Docker ( Merkel, 2014) and Singularity ( Kurtzer *et al.*, 2017).

## Genome sequence report

### Sequence data

**Figure 2.**
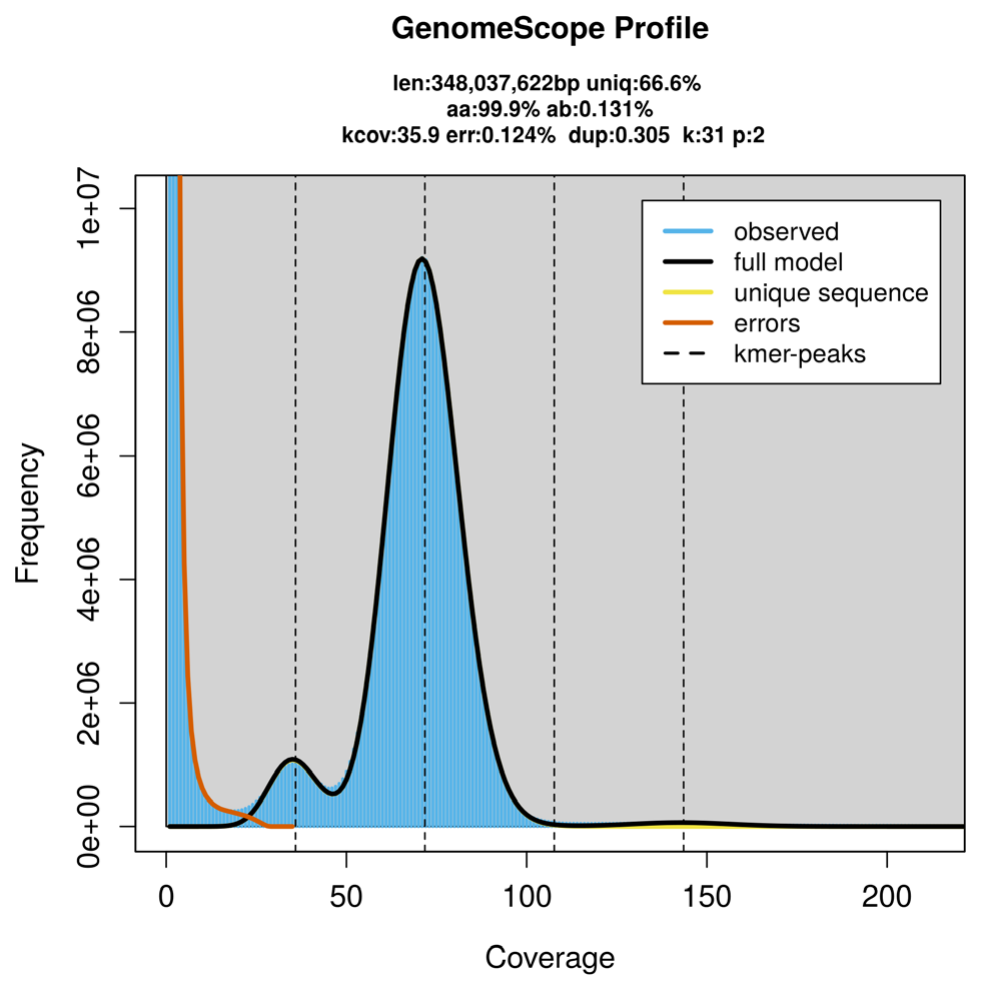
Frequency distribution of *k*-mers generated using GenomeScope2. The plot shows observed and modelled *k*-mer spectra, providing estimates of genome size, heterozygosity, and repeat content based on unassembled sequencing reads.

The genome of a specimen of *Bombus monticola* was sequenced using Pacific Biosciences single-molecule HiFi long reads, generating 26.04 Gb (gigabases) from 2.52 million reads, which were used to assemble the genome. GenomeScope2.0 analysis estimated the haploid genome size at 348.04 Mb, with a heterozygosity of 0.13% and repeat content of 33.44% ( Figure 2). These estimates guided expectations for the assembly. Based on the estimated genome size, the sequencing data provided approximately 72× coverage. Hi-C sequencing produced 154.66 Gb from 1 024.24 million reads, which were used to scaffold the assembly. RNA sequencing data were also generated and are available in public sequence repositories. Table 1 summarises the specimen and sequencing details.

**Table 1.** Specimen and sequencing data for BioProject PRJEB66738.

Platform	PacBio HiFi	Hi-C	RNA-seq
**ToLID**	iyBomMont4	iyBomMont4	iyBomMont4
**Specimen ID**	SAN00002545	SAN00002545	SAN00002545
**BioSample (source individual)**	SAMEA112198363	SAMEA112198363	SAMEA112198363
**BioSample (tissue)**	SAMEA112198386	SAMEA112198385	SAMEA112198386
**Tissue**	thorax	head	thorax
**Sequencing platform and model**	Sequel IIe	Illumina NovaSeq 6000	Illumina NovaSeq 6000
**Run accessions**	ERR12102450	ERR12102409	ERR12102410
**Read count total**	2.52 million	1 024.24 million	91.53 million
**Base count total**	26.04 Gb	154.66 Gb	13.82 Gb

### Assembly statistics

The primary haplotype was assembled, and contigs corresponding to an alternate haplotype were also deposited in INSDC databases. The final assembly has a total length of 306.99 Mb in 216 scaffolds, with 135 gaps, and a scaffold N50 of 17.95 Mb ( Table 2).

**Table 2.** Genome assembly statistics.

**Assembly name**	iyBomMont4.1
**Assembly accession**	GCA_965178685.1
**Alternate haplotype accession**	GCA_965178715.1
**Assembly level**	chromosome
**Span (Mb)**	306.99
**Number of chromosomes**	18
**Number of contigs**	351
**Contig N50**	2.89 Mb
**Number of scaffolds**	216
**Scaffold N50**	17.95 Mb
**Organelles**	Mitochondrial genome: 24.12 kb

**Figure 3.**
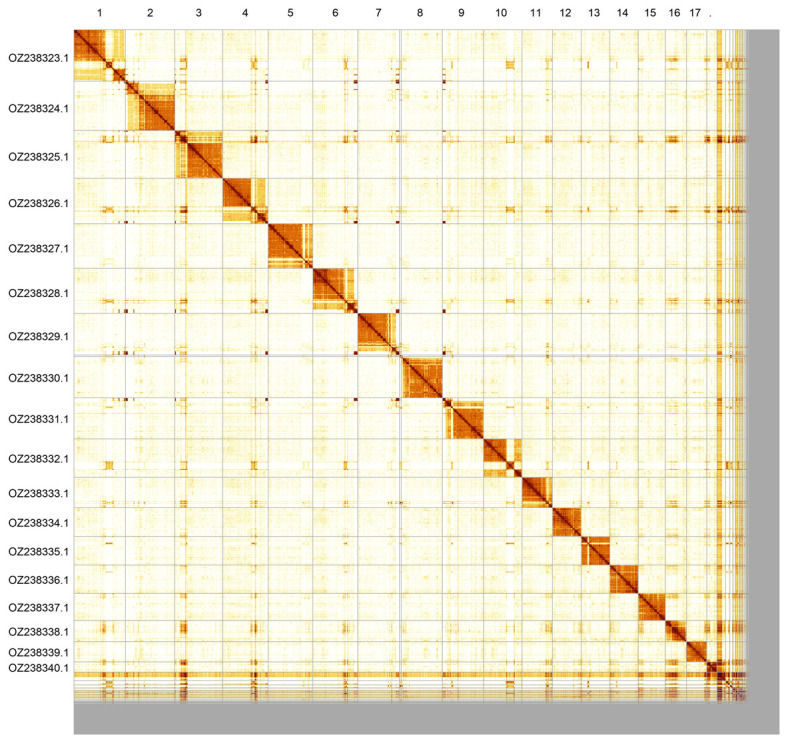
Hi-C contact map of the *Bombus monticola* genome assembly. Assembled chromosomes are shown in order of size and labelled along the axes. The plot was generated using PretextSnapshot.

Most of the assembly sequence (90.92%) was assigned to 18 chromosomal-level scaffolds. These chromosome-level scaffolds, confirmed by Hi-C data, are named according to size ( Figure 3; Table 3). The mitochondrial genome was also assembled. This sequence is included as a contig in the multifasta file of the genome submission and as a standalone record.

**Table 3.** Chromosomal pseudomolecules in the primary genome assembly of *Bombus monticola* iyBomMont4.

INSDC accession	Molecule	Length (Mb)	GC%
OZ238323.1	1	22.57	37
OZ238324.1	2	21.45	37.50
OZ238325.1	3	20.78	38
OZ238326.1	4	19.73	39
OZ238327.1	5	19.52	37
OZ238328.1	6	19.50	38
OZ238329.1	7	18.16	37
OZ238330.1	8	17.95	37
OZ238331.1	9	17.87	37.50
OZ238332.1	10	16.73	36.50
OZ238333.1	11	13.30	39
OZ238334.1	12	12.49	39
OZ238335.1	13	12.44	38
OZ238336.1	14	12.31	38
OZ238337.1	15	11.86	38
OZ238338.1	16	9.14	38
OZ238339.1	17	8.86	37.50
OZ238340.1	18	4.47	40
OZ238341.1	MT	0.02	11

### Assembly quality metrics

**Figure 4.**
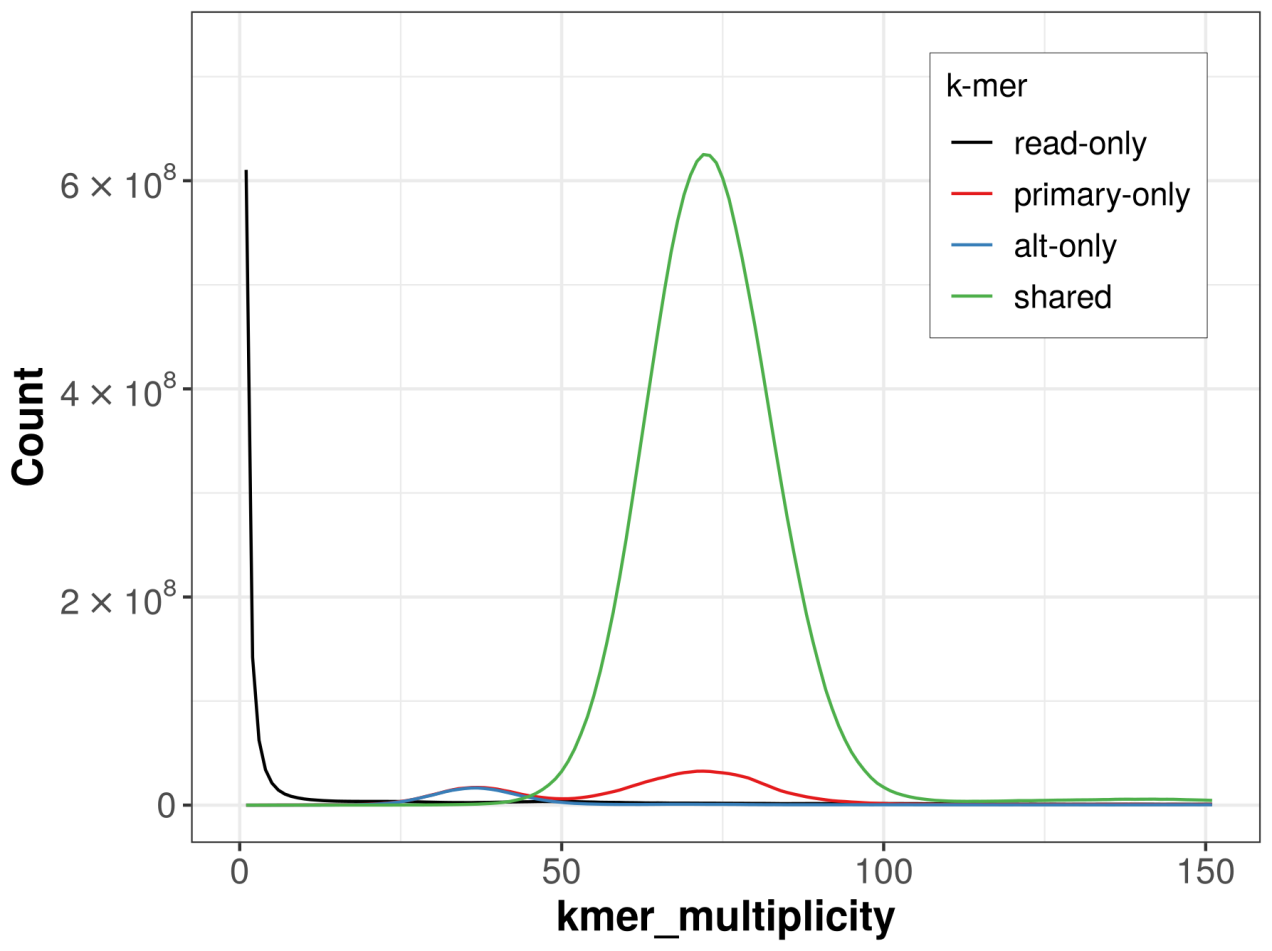
Evaluation of *k*-mer completeness using MerquryFK. This plot illustrates the recovery of *k*-mers from the original read data in the final assemblies. The horizontal axis represents *k*-mer multiplicity, and the vertical axis shows the number of *k*-mers. The black curve represents *k*-mers that appear in the reads but are not assembled. The green curve (the homozygous peak) corresponds to *k*-mers shared by both haplotypes and the red and blue curves (the heterozygous peaks) show *k*-mers found only in one of the haplotypes.

**Figure 5.**
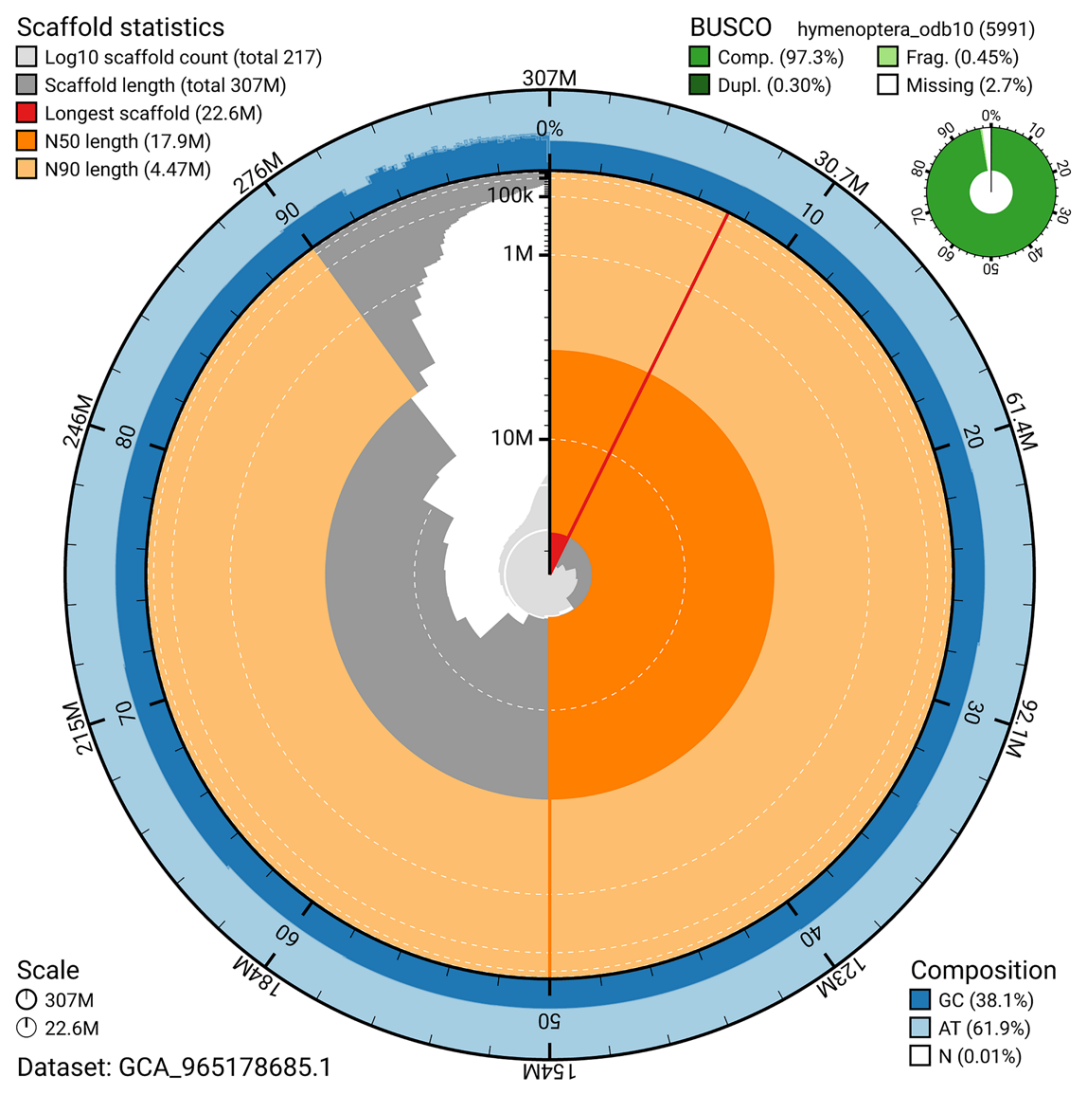
Assembly metrics for iyBomMont4.1. The BlobToolKit snail plot provides an overview of assembly metrics and BUSCO gene completeness. The circumference represents the length of the whole genome sequence, and the main plot is divided into 1,000 bins around the circumference. The outermost blue tracks display the distribution of GC, AT, and N percentages across the bins. Scaffolds are arranged clockwise from longest to shortest and are depicted in dark grey. The longest scaffold is indicated by the red arc, and the deeper orange and pale orange arcs represent the N50 and N90 lengths. A light grey spiral at the centre shows the cumulative scaffold count on a logarithmic scale. A summary of complete, fragmented, duplicated, and missing BUSCO genes in the hymenoptera_odb10 set is presented at the top right. An interactive version of this figure can be accessed on the BlobToolKit viewer.

**Figure 6.**
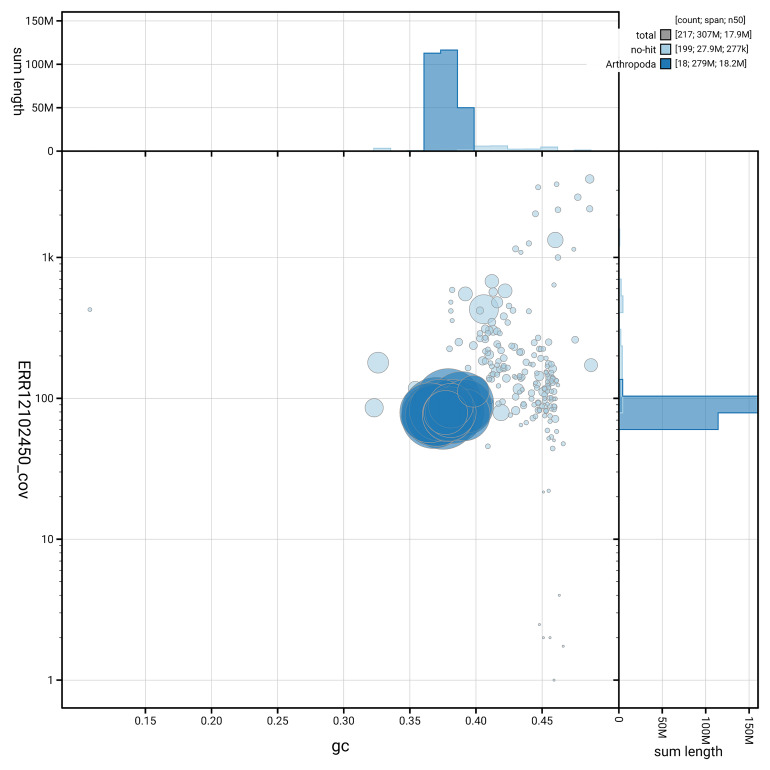
BlobToolKit GC-coverage plot for iyBomMont4.1. Blob plot showing sequence coverage (vertical axis) and GC content (horizontal axis). The circles represent scaffolds, with the size proportional to scaffold length and the colour representing phylum membership. The histograms along the axes display the total length of sequences distributed across different levels of coverage and GC content. An interactive version of this figure is available on the BlobToolKit viewer.

The combined primary and alternate assemblies achieve an estimated QV of 62.8. The *k*-mer completeness is 94.51 for the primary assembly, 89.61% for the alternate haplotype, and 97.83% for the combined assemblies ( Figure 4). BUSCO v.5.7.1 analysis using the hymenoptera_odb10 reference set ( *n* = 5 991) identified 97.3% of the expected gene set (single = 97%, duplicated = 0.3%). The snail plot in Figure 5 summarises the scaffold length distribution and other assembly statistics for the primary assembly. The blob plot in Figure 6 shows the distribution of scaffolds by GC proportion and coverage.

Table 4 lists the assembly metric benchmarks adapted from Rhie *et al.* (2021) the Earth BioGenome Project Report on Assembly Standards September 2024. The EBP metric, calculated for the primary assembly, is **6.C.Q62**, meeting the recommended reference standard.

**Table 4.** Earth Biogenome Project summary metrics for the *Bombus monticola* assembly.

Measure	Value	Benchmark
EBP summary (primary)	6.C.Q62	6.C.Q40
Contig N50 length	2.89 Mb	≥ 1 Mb
Scaffold N50 length	17.95 Mb	= chromosome N50
Consensus quality (QV)	Primary: 62.1; alternate: 63.7; combined: 62.8	≥ 40
*k*-mer completeness	Primary: 94.51%; alternate: 89.61%; combined: 97.83%	≥ 95%
BUSCO	C:97.3%[S:97.0%;D:0.3%]; F:0.5%; M:2.3%; n:5 991	S > 90%; D < 5%
Percentage of assembly assigned to chromosomes	90.92%	≥ 90%

### Wellcome Sanger Institute – Legal and Governance

The materials that have contributed to this genome note have been supplied by a Darwin Tree of Life Partner. The submission of materials by a Darwin Tree of Life Partner is subject to the **‘Darwin Tree of Life Project Sampling Code of Practice’**, which can be found in full on the [Darwin Tree of Life website] ( https://www.darwintreeoflife.org/project-resources/). By agreeing with and signing up to the Sampling Code of Practice, the Darwin Tree of Life Partner agrees they will meet the legal and ethical requirements and standards set out within this document in respect of all samples acquired for, and supplied to, the Darwin Tree of Life Project. Further, the Wellcome Sanger Institute employs a process whereby due diligence is carried out proportionate to the nature of the materials themselves, and the circumstances under which they have been/are to be collected and provided for use. The purpose of this is to address and mitigate any potential legal and/or ethical implications of receipt and use of the materials as part of the research project, and to ensure that in doing so we align with best practice wherever possible. The overarching areas of consideration are:

- Ethical review of provenance and sourcing of the material
- Legality of collection, transfer and use (national and international)

Each transfer of samples is further undertaken according to a Research Collaboration Agreement or Material Transfer Agreement entered into by the Darwin Tree of Life Partner, Genome Research Limited (operating as the Wellcome Sanger Institute), and in some circumstances, other Darwin Tree of Life collaborators.

## Data Availability

European Nucleotide Archive: Bombus monticola (mountain bumblebee). Accession number PRJEB66738. The genome sequence is released openly for reuse. The *Bombus monticola* genome sequencing initiative is part of the Darwin Tree of Life Project (PRJEB40665) and the Sanger Institute Tree of Life Programme (PRJEB43745). All raw sequence data and the assembly have been deposited in INSDC databases. The genome will be annotated using available RNA-Seq data and presented through the Ensembl pipeline at the European Bioinformatics Institute. Raw data and assembly accession identifiers are reported in Table 1 and Table 2. Pipelines used for genome assembly at the WSI Tree of Life are available at https://pipelines.tol.sanger.ac.uk/pipelines. Table 5 lists software versions used in this study.

## Author information

   •   Members of the Wellcome Sanger Institute Tree of Life Management, Samples and Laboratory team.

   •   Members of Wellcome Sanger Institute Scientific Operations – Sequencing Operations

   •   Members of the Wellcome Sanger Institute Tree of Life Core Informatics team

   •   Members of the Tree of Life Core Informatics collective

   •   Members of the Darwin Tree of Life Consortium
