# Supplementary material for: The genome sequence of the Mountain Bumble Bee, Bombus monticola Smith, 1849 (Hymenoptera: Apidae)
Source: Wellcome Open Res. 2025 Jul 25;10:367. [Version 1] doi: 10.12688/wellcomeopenres.24577.1 (PMC12409256; doi:10.12688/wellcomeopenres.24577.1)
Supplement: Peer review history

# Reviewer report: Reviewer response for version 1

**DOI:** 10.21956/wellcomeopenres.27086.r128963

**Published:** 2025-09-03

**Authors:** Leonardo Tresoldi Gonçalves

**Affiliations:** 1 Universidade Federal do Rio Grande do Sul, Porto Alegre, State of Rio Grande do Sul, Brazil

This paper reports a chromosomal-level assembly for the Mountain Bumble Bee, Bombus monticola. The methodology and results are robust and well-documented. I understand that this is supposed to be a straightforward genome report, but it would be nice if the authors compared the metrics of their assembly with other bumblebee genomes.

Are sufficient details of methods and materials provided to allow replication by others?

Yes

Is the rationale for creating the dataset(s) clearly described?

Yes

Are the datasets clearly presented in a useable and accessible format?

Yes

Are the protocols appropriate and is the work technically sound?

Yes

Reviewer Expertise:

Phylogenetics, genomics, and molecular evolution

I confirm that I have read this submission and believe that I have an appropriate level of expertise to confirm that it is of an acceptable scientific standard.

# Reviewer report: Reviewer response for version 1

**DOI:** 10.21956/wellcomeopenres.27086.r128957

**Published:** 2025-08-25

**Authors:** Cheng Sun

**Affiliations:** 1 Capital Normal University, Beijing, Beijing, China

The current study produced a chromosome-level of genome assembly for the Mountain Bumble Bee *Bombus monticola *using sample from a single female specimen. The assembled genome has 18 chromosomes, with a total length of 307 Mb. In general, the methods are clearly described and the results are sounds.

 I do not quite understand one point: are genomic DNA and RNA both extracted from the single thorax of the same female?

Are sufficient details of methods and materials provided to allow replication by others?

Yes

Is the rationale for creating the dataset(s) clearly described?

Yes

Are the datasets clearly presented in a useable and accessible format?

Yes

Are the protocols appropriate and is the work technically sound?

Yes

Reviewer Expertise:

bumblebee genome evolution

I confirm that I have read this submission and believe that I have an appropriate level of expertise to confirm that it is of an acceptable scientific standard.
